# Diagnostic accuracy of chip hybridization from formalin-fixed paraffin embedded bioptic specimens in a cohort with predominantly extrapulmonary mycobacterial disease

**DOI:** 10.1186/s12866-026-04875-2

**Published:** 2026-03-07

**Authors:** Ria Winkelmann, Belana Blasczok, Melanie Winter, Peter Wild, Maria J.G.T. Vehreschild, Volkhard A.J. Kempf, Thomas A. Wichelhaus, Raja Idris, Nils Wetzstein

**Affiliations:** 1 Dr. Senckenberg Institute for Pathology and Human Genetics, Goethe University Frankfurt, Frankfurt am Main, Germany; 2 Department II of Internal Medicine, Infectious Diseases, Goethe University Frankfurt, University Hospital, Frankfurt am Main, Germany; 3 Mycobacterial Infection Research Unit (MIRU), Goethe University Frankfurt, Frankfurt am Main, Germany; 4 Institute of Medical Microbiology and Infection Control, Goethe University Frankfurt, University Hospital, Frankfurt am Main, Germany; 5 Molecular and Experimental Mycobacteriology, Research Center Borstel, Borstel, Germany; 6 University Hospital Frankfurt, Theodor-Stern-Kai 7, Frankfurt am Main, 60590 Germany

**Keywords:** TB, Tuberculosis, NTM, Non-tuberculous mycobacteria, Chip hybridization

## Abstract

**Introduction:**

Extrapulmonary tuberculosis (TB) and non-tuberculous mycobacterial (NTM) disease remain a diagnostic challenge and conventional methods such as mycobacterial culture or molecular methods (e.g. GeneXpert) performed on formalin-fixed paraffin embedded (FFPE) specimens are often unsuccessful. In this study, we assessed the diagnostic accuracy of chip hybridization from FFPE specimens in a cohort with predominantly extrapulmonary mycobacterial disease.

**Methods:**

FFPE specimens from patients with presumed mycobacterial disease underwent chip hybridization with two different assays (MYCO Direct 1.7 and MYCO Chip Vision Array 2.0) in addition to conventional diagnostic methods for TB and NTM disease including mycobacterial culture, molecular methods and conventional histology. The different techniques were assessed with regards to sensitivity, specificity and species identification.

**Results:**

Overall, 184 samples were assessed by one of the two chip hybridization techniques (MYCO Direct 1.7 n=65 and MYCO Chip Vision Array n=119). Samples were mainly extrapulmonary (160/184, 87.0%). Of all samples, 32 (17.4%) turned positive in chip hybridization and 74/184 patients (40.2%) were diagnosed with clinically relevant mycobacterial disease (59 with TB and 15 with NTM disease). For the detection of *Mycobacterium tuberculosis* complex (MTBC), sensitivity was 38.9% when compared to mycobacterial culture from the same specimen. For the detection of NTM, sensitivity was 50% against NTM culture. Species identification was overall consistent with conventional methods but did not allow for species differentiation of members of the MTBC or *Mycobacterium avium* complex, respectively.

**Conclusion:**

As extrapulmonary TB and NTM disease remain a diagnostic challenge, the investigated chip hybridization techniques might present a complementation of the diagnostic portfolio. However, with a low sensitivity to detect MTB from extrapulmonary FFPE specimens, they should not supplant conventional diagnostic methods but could be used to rule in TB when native specimens are not available.

**Supplementary Information:**

The online version contains supplementary material available at 10.1186/s12866-026-04875-2.

## Introduction

Mycobacterial diseases encompass all infections caused by members of the genus *Mycobacterium* which now comprises more than 200 species [1]⁠.

Tuberculosis (TB) is caused by the *Mycobacterium tuberculosis* complex (MTBC) and leads to the highest number of deaths caused by a single agent in the world. In 2024, 10.7 million people suffered from TB disease and 1.23 million people died from it [2]⁠. While the majority of patients suffer from pulmonary TB, approximately 25% of patients worldwide experience extrapulmonary TB (EPTB) [2]⁠. The geographical distribution of EPTB is variable with people from the WHO Eastern Mediterranean and the South-East Asia Region being most affected [3, 4]. However, EPTB poses a significant diagnostic challenge.

Non-tuberculous mycobacteria (NTM) comprise all mycobacteria except the MTBC, *M. leprae* and *M. lepromatosis* [5]. These pathogens are ubiquitous and can be isolated from different environmental sources, such as dust, household water or soil [6, 7]. Under certain circumstances they may cause clinically relevant disease similar to TB. The most frequent manifestation of clinically relevant NTM-infection is pulmonary disease, but extrapulmonary and disseminated infections can also occur, especially in patients with immunosuppression (e.g. HIV infection) [8, 9].

For TB and NTM infection, mycobacterial culture with a specific medium (e.g. Löwenstein-Jensen or Middlebrook agar) is still the diagnostic gold standard [10]. However, the WHO endorses the wide use of molecular methods, such as GeneXpert technology instead of cultivating the pathogen, especially in high incidence settings of TB with limited laboratory resources [11]. In a high income, low incidence setting for TB (such as Germany), the diagnosis is often based on a spectrum of methods including culture, molecular techniques for pathogen identification (including GeneXpert), histology (with typical histological hallmarks of mycobacterial disease), smear microscopy (e.g. Auramine, Kinyoun or Ziehl-Neelsen stain) and clinical presentation [12].

In this low incidence setting, EPTB might often be mistaken for malignant disease [13]. Therefore, tissue extraction and subsequent pathological examination is frequently the first step in the diagnostic cascade. Histological hallmarks of mycobacterial disease in tissue specimens are necrosis, caseating epithelioid granuloma, but also the identification of acid-fast bacilli (AFB) [14, 15]. However, from these materials, a bacterial culture is often not possible anymore [16]. Of note, conventional molecular methods such as GeneXpert MTB/RIF often show negative results when performed on formalin-fixed paraffin embedded (FFPE) raw material [17–19]. DNA fragmentation, cross-linking, chemical modifications, and PCR inhibition are the principal technical barriers to molecular analysis of FFPE specimens, necessitating specialized extraction protocols and assay designs that accommodate short, damaged nucleic acid templates [20–22]. To provide an additional option for the diagnosis of mycobacterial diseases, chip hybridization assays have been tested on these valuable specimens [23, 24]. These also include a PCR amplification step of extracted DNA, but due to the small amplicon sizes, the assays are supposed to be better suited for the analysis of FFPE tissue sections. Most assays allow for the identification of different mycobacterial species including the MTBC, but also a variety of NTM-species, including clinically relevant species such as the *M. avium* complex (MAC), *M. kansasii*,* M. xenopi*,* M. abscessus* or *M. marinum*. However, the diagnostic accuracy of these assays in a real-world setting remains largely uninvestigated.

In this study, we evaluate the diagnostic possibilities of two different chip hybridization assays (MYCO Chip Vision array 2.0 and MYCO direct 1.7) in the diagnosis of clinically relevant mycobacterial disease including TB and NTM disease from FFPE tissue specimens in comparison to conventional methods that are used as a gold standard.

## Methods

### Included patients

All patients with suspected mycobacterial disease treated at the University Hospital Frankfurt, Germany, who had undergone one of two chip hybridization techniques for mycobacteria between 2019 and 2024 were included into the study. Clinical data was retrieved from our local patient information system (ORBIS, Dedalus Healthcare, Bonn, Germany). This included basic demographic data, geographical origin stratified by WHO regions [25], and basic clinical characteristics.

As all samples were acquired during clinical routine and this was a retrospective study, informed consent was waived by the Institutional Review Board (ethics committee) of the University Hospital Frankfurt and the study approved under file numbers 2022 − 672_3 and 2023 − 570_3.

### Chip hybridization technique

Pathological specimens were collected during clinical routine care at the University Hospital Frankfurt. DNA was extracted from FFPE tissue specimens using either the RNeasy Micro Kit, Qiagen, Hilden, Germany (2019–2023) or Maxwell^®^ RSC DNA FFPE Kit (since 2023) (Promega, Madison, Wisconsin, USA). DNA concentration was assessed by a Qubit 4 Fluorometer (Thermo Fisher Scientific Inc., Waltham, Massachusetts, USA). Chip hybridization was performed either with the MYCO Chip Vision array 2.0 (Zytovision, Berlin, Germany; since 2023) or the MYCO Direct 1.7, (Chipron, Berlin, Germany; 2019–2022) according to the manufacturer’s recommendations. Species identification with the MYCO Chip Vision array 2.0 includes the MTBC, *M. abscessus*, MAC, *M. chelonae*,* M. chimaera*,* M. fortuitum*,* M. genavense*,* M. gordonae*,* M. haemophilum*,* M. kansasii*,* M. malmoense*,* M. marinum/ulcerans*,* M. scrofulaceum/M. parascrofulaceum*,* M. simiae*,* M. smegmatis*,* M. szulgai*, and *M. xenopi.* The MYCO Direct 1.7 includes three genus specific probes (Mycobacteria Genus I to III) and further allows the species identification of the MTBC, MAC, *M. kansasii*,* M. xenopi*,* M. abscessus*,* M. gordonae*,* M. peregrinum*,* M. szulgai*,* M. haemophilum*,* M. marinum/ulcerans*,* M. simiae*, and *M. smegmatis.*

### Histological procedures

Further, all FFPE specimens underwent histological routine analysis at the Dr. Senckenberg Institutes of Pathology and Human Genetics, Frankfurt, Germany. Routine diagnostics included fixation in 4% buffered formalin, paraffin embedding and routine Hematoxylin and eosin (H&E) staining. In case of histopathological (caseating necrosis, epithelioid cell granulomas, or giant cells) and/or clinical suspicion, samples were stained via Ziehl-Neelsen technique according to manufacturers’ instructions (*figure *S1) (Artisan™ Acid-Fast Bacillus (AFB) Stain Kit, Agilent, Santa Clara, CA, USA).

### Microbiological procedures

Procedures to diagnose TB or NTM disease included mycobacterial culture from native specimens (using Löwenstein-Jensen-agar, Stonebrink-agar and mycobacterial growth indicator tube - MGIT) at 37 °C for eight weeks and molecular methods from native specimens (16 S rDNA PCR from non-respiratory material, Genotype CMdirect, Hain/Bruker, Nehren, Germany, primarily from respiratory materials, and GeneXpert from any native material in case of suspicion for TB), as well as from culture isolates for species identification as previously described (internal transcribed spacer - ITS-PCR) [10, 11]. For the MTBC, genotypic drug susceptibility testing was performed for rifampicin with GeneXpert (Cepheid, Sunnyvale, USA) and for isoniazid/rifampicin with Genotype MTBDRplus (Hain/Bruker, Nehren, Germany). Microscopy was performed using either Auramine (ELITech Group/Gonotec GmbH, Berlin Germany) or Kinyoun stain (Carbol fuchsin – Sigma Aldrich/Merck, Darmstadt, Germany; Acid alcohol – Roth, Karlsruhe, Germany; methylene blue – AppliChem, Darmstadt Germany) on native materials. These assays were performed under strict quality-controlled criteria (laboratory accreditation according to ISO 15189:2011 standards; certificate number D–ML–13102–01–00) at the Institute for Medical Microbiology and Infection Control, University Hospital Frankfurt, Germany. These microbiological procedures as well as standard histology were defined as “conventional methods”.

### Case definitions

Clinically relevant mycobacterial disease was defined as a clinical, microbiological, histological or combined diagnosis of TB or NTM disease leading to the intention or initiation of antimycobacterial therapy. For all patients meeting these criteria, detailed clinical characteristics were recorded: clinical form of the disease (pulmonary, extrapulmonary, or disseminated), organs affected by mycobacterial infection, relevant comorbidities, predisposing factors and outcome. According to WHO definitions, pulmonary disease was defined as affection of the lung parenchyma or the tracheobronchial tree, extrapulmonary disease as clinical manifestations not meeting these criteria [26]. Disseminated disease was defined as any TB or NTM infection affecting more than one body region.

### Statistical analysis

All data was analysed in R v 4.1. (“Beagle Scouts”) with packages of the *tidyverse* [27, 28]. Categorical data is depicted as numerator with denominator and percentages, continuous data as mean with range for normally distributed data, and median with interquartile range for non-normally distributed data. Normality was tested using the Shapiro-Wilk test. Logistic regression to identify potential predictors for a positive chip hybridization assay was performed using the *finalfit* package within R [29]. Sensitivity for the hybridization tests was assessed against the diagnostic gold standard – mycobacterial culture – for all samples for which both procedures had been performed. Here, another aliquot of the native specimen was used for conventional methods. For all statistical tests a significance level of alpha = 0.05 was used.

## Results

### Samples examined by chip hybridization and histology

Overall, 184 specimens were assessed by one of the two chip hybridization techniques (Table 1). The majority of specimens were extrapulmonary (160/184, 87.0%). The most frequent sample types were lymph nodes (*n* = 72), followed by tracheobronchial biopsies (*n* = 24), soft tissue or abdominal biopsies (*n* = 17 each). In total, 65 specimens were examined with the MYCO Chip Vision array 2.0 and 119 with the MYCO Direct 1.7.

**Table 1:** General characteristics of retrieved specimens

	Both methods *n* = 184	MYCO Chip Vision Array 2.0 *n* = 65	MYCO Direct 1.7 *n* = 119
	pos/*N* (%)	pos/*N* (%)	pos/*N* (%)
Samples	32/184 (17.4%)	15/65 (23.1%)	17/119 (14.3%)
Lymph node	11/72 (15.3%)	7/31 (22.6%)	4/41 (9.8%)
Soft tissue	1/17 (5.9%)	1/5 (20.0%)	0/12 (0.0%)
Bone / Bone Marrow / Spine	0/9 (0.0%)	0/1 (0.0%)	0/8 (0.0%)
Tracheobronchial	6/24 (25.0%)	3/11 (27.3%)	3/13 (23.1%)
Abdominal	3/17 (17.6%)	0/5 (0.0%)	3/12 (25.0%)
Gastrointestinal	5/11 (45.5%)	1/2 (50.0%)	4/9 (44.4%)
Muscle	0/2 (0.0%)	0/1 (0.0%)	0/1 (0.0%)
Pleura	2/6 (33.3%)	1/2 (50.0%)	1/4 (25.0%)
Urogenital	1/5 (20.0%)	1/2 (50.0%)	0/3 (0.0%)
Immune system	0/2 (0.0%)	0/2 (0.0%)	0/0 (0.0%)
Other	3/19 (15.8%)	1/3 (33.3%)	2/16 (12.5%)
Detected species Chip Hybridization			
* M. tuberculosis*	16/32 (50.0%)	6/15 (40.0%)	10/17 (58.8%)
MAC	4/32 (12.5%)	3/15 (20.0%)	1/17 (5.9%)
* M. gordonae*	2/32 (6.3%)	2/15 (13.3%)	0/17 (0.0%)
* M. xenopi*	2/32 (6.3%)	2/15 (13.3%)	0/17 (0.0%)
* M. tuberculosis / M. xenopi*	1/32 (3.1%)	1/15 (6.7%)	0/17 (0.0%)
MAC */ M. malmoense*	1/32 (3.1%)	1/15 (6.7%)	0/17 (0.0%)
Genus III	3/32 (9.4%)	0/15 (0.0%)	3/17 (17.6%)
Genus I + III	2/32 (6.3%)	0/15 (0.0%)	2/17 (11.8%)
Histological features			
Ziehl-Neelsen in Pathology	13/184 (7.1%)	4/65 (6.2%)	9/119 (7.6%)
Necrosis	78/184 (42.4%)	32/65 (49.2%)	46/119 (38.7%)
Abscess-forming Granulocytes	33/184 (17.9%)	11/65 (16.9%)	22/119 (18.5%)
Chronic lymphocytes	36/184 (19.6%)	13/65 (20.0%)	23/119 (19.3%)
Granulating Fibrosis	10/184 (5.4%)	5/65 (7.7%)	5/119 (4.2%)
Epithelioid granulomas	68/184 (37.0%)	28/65 (43.1%)	40/119 (33.6%)

Out of all samples, 32 were positive for any mycobacterial species in the chip hybridization assay (17.4%, *figure S2*). MYCO Chip Vision array 2.0 resulted in slightly more positive results than the MYCO Direct 1.7 (15/65, 23.1% vs. 17/119, 14.3%, *p* = 0.13). The MTBC was the species group most frequently identified (16/32, 50%), followed by MAC (*n* = 4), *M. gordonae* and *M. xenopi* (*n* = 2 each). In two samples, two different mycobacterial species were detected (*M. tuberculosis/M. xenopi* and MAC/*M. malmoense*, respectively). With MYCO Direct 1.7, no mixed infections were detected.

Most frequent histological features were necrosis (78/184, 42.4%), epithelioid granulomas (68/184, 43.1%) and chronic lymphocyte infiltration (36/184, 20%). AFB were seen by the pathologist in 13/184 specimens (7.1%).

Patient age or gender, DNA concentration, specimen type or different histological features were not significant positive predictors for a positive chip hybridization test. However, a typical histology with epithelioid granulomas and necrosis increased the odds for a positive result (OR = 1.92, *p* = 0.096).

### Characteristics of patients with clinically relevant mycobacterial disease

In total, 74/184 patients (40.2%) were diagnosed with clinically relevant mycobacterial disease (Table 2). The majority of those patients were male (54.1%). A history of migration was observed in 60.8% (*n* = 45), but most of these patients originated from the WHO European Region 14/45 (31.1%). Overall, 36 of 57 (63.2%) available interferon gamma release assay (IGRA) results were positive. Most frequent predisposing factors were HIV (18.9%), immunosuppressive therapy (18.9%), malignancy (13.5%) and chronic vascular disease (13.5%). Nearly a third (27%) of patients were smokers.

**Table 2:** Baseline characteristics of patients with diagnosed clinically relevant mycobacterial disease

	All *n* = 74	TB *n* = 59	NTM *n* = 15
	*n*/*N* (%)	*n*/*N* (%)	*n*/*N* (%)
Age	Median 36 years	Median 34 years	Median 51 years
Sex			
Female	34/74 (45.9%)	30/59 (50.8%)	4/15 (26.7%)
male	40/74 (54.1%)	29/59 (49.2%)	11/15 (73.3%)
Migration history	45/74 (60.8%)	42/59 (71.2%)	3/15 (20.0%)
WHO Region			
African Region	8/45 (17.8%)	8/42 (19.0%)	0/3 (0.0%)
Region of the Americas	1/45 (2.2%)	1/42 (2.4%)	0/3 (0.0%)
Eastern Mediterranean Region	10/45 (22.2%)	9/42 (21.4%)	1/3 (33.3%)
European Region	41/45 (31.1%)	12/42 (28.6%)	2/3 (66.7%)
South-East Asia Region	7/45 (15.6%)	7/42 (16.7%)	0/3 (0.0%)
Western Pacific Region	5/45 (11.1%)	5/42 (11.9%)	0/3 (0.0%)
IGRA positive	36/57 (63.2%)	36/49 (73.5%)	0/8 (0.0%)
Comorbidities			
HIV	14/74 (18.9%)	4/59 (6.8%)	10/15 (66.7%)
Immunosuppressive	14/74 (18.9%)	9/59 (15.3%)	5/15 (33.3%)
Diabetes	2/74 (2.7%)	2/59 (3.4%)	0/15 (0.0%)
Malignancy	10/74 (13.5%)	6/59 (10.2%)	4/15 (26.7%)
CVD	10/74 (13.5%)	10/59 (16.9%)	0/15 (0.0%)
Smoker	20/74 (27.0%)	14/59 (23.7%)	6/15 (40.0%)
CKD	4/74 (5.4%)	3/59 (5.1%)	1/15 (6.7%)
Clinical form of mycobacterial disease			
Pulmonary	6/74 (8.1%)	6/59 (10.2%)	0/15 (0.0%)
Extrapulmonary	41/74 (55.4%)	33/59 (55.9%)	8/15 (53.3%)
Disseminated	27/74 (36.5%)	20/59 (33.9%)	7/15 (46.7%)
Organs affected			
Lung	25/74 (33.8%)	19/59 (32.2%)	6/15 (40.0%)
Pleura	7/74 (9.5%)	6/59 (10.2%)	1/15 (6.7%)
Lymph node	45/74 (60.8%)	35/59 (59.3%)	10/15 (66.7%)
Abdominal	12/74 (16.2%)	9/59 (15.3%)	3/15 (20.0%)
Bone	6/74 (8.1%)	3/59 (5.1%)	3/15 (20.0%)
Urogenital	7/74 (9.5%)	7/59 (11.9%)	0/15 (0.0%)
CNS	2/74 (2.7%)	1/59 (1.7%)	1/15 (6.7%)
Spine	6/74 (8.1%)	5/59 (8.5%)	1/15 (6.7%)
Miliary	5/74 (6.8%)	5/59 (8.5%)	0/15 (0.0%)
Other organs	5/74 (6.8%)	3/59 (5.1%)	2/15 (13.3%)
Deceased	3/74 (4.1%)	3/59 (5.1%)	0/15 (0.0%)

Of patients with clinically relevant mycobacterial disease, 59 were diagnosed with TB (79.7%) and 15 with relevant NTM-infection (20.3%). Most patients suffered from extrapulmonary or disseminated disease (55.4% and 36.5%), with 60.8% of patients showing signs of lymph node affection and 33.8% affection of the lung. Specifically, 33.9% of patients with TB had disseminated disease, while 46.7% of patients with NTM-disease suffered from disseminated infections. Of all patients, 4.1% of patients died during the observation period.

Patients with TB were younger than patients with NTM disease (median 34 years vs. median 51 years) and more frequently migrated (71.2% vs. 20%). Two thirds of patients with NTM disease were HIV positive (66.7%).

### Comparison of chip hybridization and conventional methods

**Fig. 1:**
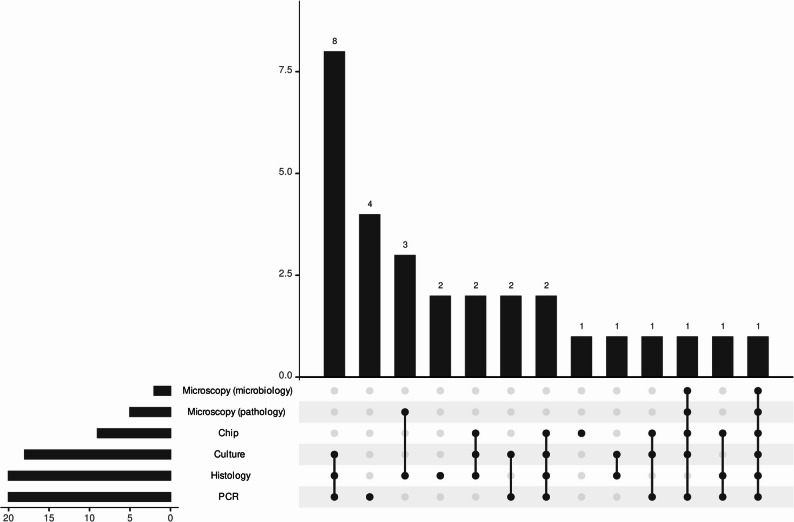
Upset plot of positivity of different diagnostic methods from the same material for patients with diagnosed TB disease. Left panel shows overall number of samples that are positive by diagnostic modality. Right upper panel shows the number of samples that are positive in a given combination of diagnostic modalities (right lower panel). Microscopy refers to the detection of acid-fast bacilli (either performed by a microbiologist or pathologist)

In 27/74 patients with clinically relevant mycobacterial disease, the chip hybridization showed a positive result (36.5%). In 44/74 patients, another aliquot of the same material was used to perform either mycobacterial culture (*n* = 43), PCR (GeneXpert/16S rDNA PCR/Genotype CMdirect) (*n* = 40) or microscopy (*n* = 39) (Fig. 1, *table *S1,* figure S3*). Among these samples, 13/44 (29.5%) were positive by chip hybridization, 22/43 (51.2%) by mycobacterial culture, 22/40 (55%) by PCR, 4/39 (10.3%) of samples displayed AFB in microscopy performed by microbiologists, and 7/32 (21.9%) of samples were observed to contain AFB by pathologists. Only two patients considered to have clinically relevant mycobacterial disease were diagnosed solely by chip hybridization (one TB disease and one NTM disease, Fig. 1, *figure S3*).

Compared to mycobacterial culture as the gold standard, sensitivity was 57.1% (4/7 culture positive samples) for the MYCO Chip Vision array 2.0 and 33.3% (5/15 culture positive samples) for MYCO Direct 1.7 (Table 3). For TB, sensitivities were 50.0% and 33.3% against MTBC culture, respectively. For NTM, sensitivities were 100% and 33.30% against NTM culture, respectively.

**Table 3:** Sensitivity and specificity of chip hybridization methods with mycobacterial culture from the same specimen as comparator

	MYCO Chip Vision array 2.0	MYCO Direct 1.7
	*n*/*N* [%]	*n*/*N* [%]
Any mycobacterial disease		
Sensitivity	4/7 (57.1%)	5/15 (33.3%)
Specificity	5/8 (62.5%)	12/13 (92.3%)
TB		
Sensitivity	3/6 (50.0%)	4/12 (33.3%)
Specificity	11/15 (73.3%)	12/13 (92.3%)
NTM		
Sensitivity	1/1 (100.0%)	1/3 (33.3%)
Specificity	13/14 (92.9%)	25/25 (100%)

**Fig. 2:**
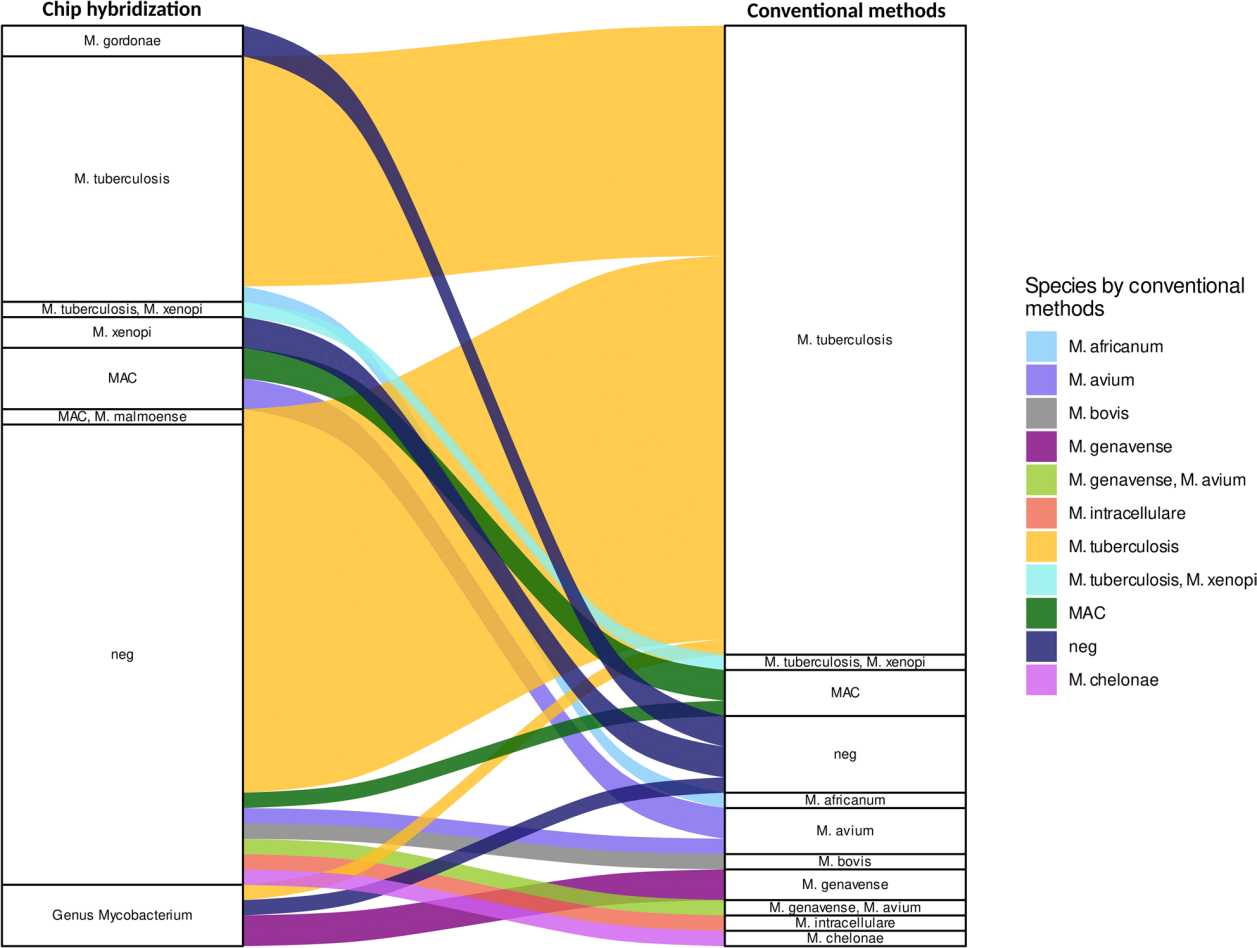
Sankey diagram of patient-based consistency of species identification by chip hybridization and conventional methods. Left panel shows species designation by chip hybridization, right panel shows the final species designation by conventional methods identified as the cause of clinically relevant disease. Cases are connected by colored lines based on species identification by conventional methods. MAC - *Mycobacterium avium* complex; neg - negative.

By conventional methods, *M. tuberculosis/M. tuberculosis* complex was the most frequent pathogen identified in 41 patients (55.4%), *M. africanum*, *M. bovis*, and *M. tuberculosis/M. xenopi* coinfection were identified in one patient each by culture (Fig. 2, *table S2*). NTM alone were identified in 11 patients (three members of the MAC without identification to species level, three *M. avium*, two *M. genavense*, one *M. intracellulare*, one *M avium/M. genavense* coinfection and one *M. chelonae/M. immunogenicum*).

In samples from patients with mycobacterial disease that were positive by conventional methods and were investigated by chip hybridization (*n* = 34), species identification by chip hybridization was consistent for NTM in 4/4 samples and for MTBC in 8/8 samples. However, in 5/34 samples NTM and 17/34 samples MTBC were identified by conventional methods only (Fig. 2). *M. genavense* was only identified to a genus level by chip hybridization while *M. avium* was only identified to a complex level. Further, different members of the MTBC (e.g. *M. africanum*) were not differentiated. In one case the chip hybridization identified MAC and *M. malmoense* in the chip hybridization, but *Mycobacterium tuberculosis* was found by conventional methods.

## Discussion

In this study, we present comprehensive diagnostic data from a cohort with predominantly extrapulmonary TB and NTM disease. The two different chip hybridization assays show low sensitivities but may present a complementation of conventional diagnostic methods, especially in cases where only FFPE specimens are available. However, our data suggests that they should not be used as a primary diagnostic method for the diagnosis of extrapulmonary mycobacterial disease.

Overall sensitivities for both methods were 40.9% against mycobacterial culture. Only, 29.7% of patients with relevant mycobacterial disease showed positive chip hybridization results. Mycobacterial culture still remains the gold standard for diagnosing TB. However, molecular methods, such as GeneXpert have supplanted it in many settings [11]. Sensitivity of the GeneXpert MTB/RIF for the detection of pulmonary TB is much higher than the presented figures here (88–90%) [30]. Similarly, GeneXpert allows identification of MTBC from lymph node aspirates with a reasonable sensitivity as well (> 80%) [31]. However, these methods are mainly validated and used in native materials, while the chip hybridizations assays in our study were predominantly performed in extrapulmonary FFPE specimens. When investigated in these kinds of specimens the sensitivities of chip hybridization and GeneXpert MTB/RIF become comparable [17, 32]. Nevertheless, our results underline that chip hybridization should not be used as a standard diagnostic method but can be used with care in situations in which native specimens are not available. Interestingly, our data suggests a higher sensitivity for the MYCO Chip Vision Array 2.0 versus the MYCO Direct 1.7. However, these figures have to be interpreted with care as only very few specimens were examined both in chip hybridization and in mycobacterial culture.

EPTB remains a diagnostic challenge. In clinical practice, specimens are often paraffinized and no native specimens are retrieved, especially in low incidence settings for TB and when malignancy is suspected. As no mycobacterial culture can be performed from these samples, the investigated chip hybridization methods can present a complementation of the diagnostic cascade in extrapulmonary mycobacterial disease. Interestingly, AFB were more frequently detected by pathologists than microbiologists (10.3% vs. 21.9%). It is of note that this might be linked to the fact that the subset of investigated samples is not necessarily the same.

Identification of mycobacterial species was overall consistent but in contrast to molecular methods such as the Hain test or ITS-PCR the chip hybridization assays lack species discrimination for the MAC or the MTBC. Furthermore, rare NTM species such as *M. genavense* could not be discriminated to a species level. In several cases, the hybridization assays identified NTM in patients with TB disease raising the possibility of coinfection, contamination or false positives. While the documentation of the Zytovision MYCO Chip Vision Array states a 100% specificity for detectable mycobacterial species (stated in the product manual of the used kit which can be accessed via https://www.zytovision.com/downloads_products/manuals/de/va-0005-ce-ivd-de.pdf) our results from a real world setting cannot confirm this.

Detection of DNA and RNA from FFPE tissue has been challenging due to fixation artifacts and degradation of nucleic acids, which, in case of mycobacterial infection, is aggravated by the low bacterial load [33]. The evolution of extraction methods followed the special needs of that material, especially in designing assays with short amplicon lengths [18]. Nevertheless, the use of paraffin embedded material is often associated with inferior results compared to fresh or fresh frozen tissue [34]. However, the assays tested here have the advantage of a short laboratory turnaround time and a low cost per sample. Additionally, readout is easily performed. An alternative is the use of ddPCR technique, which showed to be a sensitive and reliable method. This method, nevertheless, is not widely available, requires experienced laboratory staff and the cost per reaction does not significantly differ from standard methods [35].

This study has several limitations: first, as this is a retrospective study, not all specimens underwent both – chip hybridization and diagnostic procedures with conventional methods. Second, we cannot directly compare the sensitivity of chip hybridization techniques to those of GeneXpert or culture, as these are mainly evaluated in native materials and not in FFPE samples. Third, for samples in which both - chip hybridization and conventional methods - were performed, two different portions of the same specimen (e.g. a biopsy) were investigated, raising the possibility of discordant results. And finally, case numbers for the different disease entities remain relatively low, as well as the number of specimens in which both chip hybridization and conventional methods were performed, decreasing the interpretability of the sensitivities presented here and warranting the evaluation of these chip hybridization techniques in future studies with larger patient populations.

## Conclusion

As extrapulmonary TB and NTM disease remain a diagnostic challenge, the investigated chip hybridization techniques can present a complementation of the diagnostic cascade. They might be used to rule in TB especially when native specimens are not available. However, their low sensitivity underlines the importance of investigating native specimens with conventional diagnostic methods when mycobacterial disease is suspected.

## Supplementary Information

Supplementary Material 1.

## Data Availability

All data supporting the findings of this study are available within the paper and its Supplementary Information.
