# Supplementary material for: Diagnostic accuracy of chip hybridization from formalin-fixed paraffin embedded bioptic specimens in a cohort with predominantly extrapulmonary mycobacterial disease
Source: BMC Microbiol. 2026 Mar 7;26:372. doi: 10.1186/s12866-026-04875-2 (PMC13088699; doi:10.1186/s12866-026-04875-2)
Supplement: Supplementary file 1 — Supplementary Material 1. [file 12866_2026_4875_MOESM1_ESM.docx]

**Supplementary tables:**

Table S1: Microbiological and pathological results in patients with diagnosed mycobacterial disease.

|  |  | **All methods** | **Chip hybridization** | **Culture** | **PCR*** | **Microscopy (micro)** | **Microscopy (patho)** | **Histology** |
| --- | --- | --- | --- | --- | --- | --- | --- | --- |
|  |  | **n/N [%]** | **n/N [%]** | **n/N [%]** | **n/N [%]** | **n/N [%]** | **n/N [%]** | **n/N [%]** |
| **Same material** | | 37/44 (84.1%) | 13/44 (29.5%) | 22/43 (51.2%) | 22/40 (55.0%) | 4/39 (10.3%) | 7/32 (21.9%) | 27/44 (61.4%) |
|  | Lymph node | 25/26 (96.2%) | 6/26 (23.1%) | 15/25 (60%) | 14/24 (53.3%) | 1/22 (4.5%) | 4/18 (22.2%) | 20/26 (76.9%) |
|  | Soft tissue | 3/4 (75.0%) | 1/4 (25.0%) | 2/4 (50.0%) | 3/4 (75.0%) | 1/4 (25.0%) | 1/2 (50.0%) | 1/4 (25.0%) |
|  | Bone/Bone Marrow / Spine | 2/4 (50.0%) | 0/4 (0.0%) | 1/4 (25.0%) | 0/4 (0.0%) | 0/2 (0.0%) | 1/4 (25.0%) | 2/4 (50.0%) |
|  | Tracheobronchial | 3/3 (100.0%) | 2/3 (66.7%) | 1/3 (33.3%) | 1/3 (33.3%) | 0/3 (0.0%) | 0/2 (0.0%) | 2/3 (66.7%) |
| ‍ | Pleura | 2/2 (0%) | 2/2 (100%) | 1/2 (50%) | 0/2 (0%) | 0/2 /0%) | 0/2 (0%) | 1/2 (50%) |
|  | Abdominal | 1/2 (50.0%) | 1/2 (50.0%) | 1/1 (100.0%) | 1/2 (50.0%) | 1/2 (50.0%) | 1/2 (50.0%) | 1/2 (50.0%) |
|  | Gastrointestinal | 2/2 (100.0%) | 1/2 (50.0%) | 1/2 (50.0%) | 2/2 (100.0%) | 0/2 (0.0%) | 0/1 (0.0%) | 0/2 (0.0%) |
|  | Other | 1/1 (100.0%) | 0/1 (0.0%) | 0/1 (0.0%) | 1/1 (100.0%) | 1/1 (100.0%) | 0/1 (0.0%) | 0/1 (0.0%) |
| **Different materials** | |  |  |  |  |  |  |  |
|  | Lymph node | 11/20 (55.0%) | -- | 5/20 (25.0%) | 6/20 (30.0%) | 4/20 (20.0%) | 4/20 (20.0%) | 10/20 (50.0%) |
|  | Bone marrow | 2/5 (40.0%) | -- | 0/5 (0.0%) | 0/5 (0.0%) | 0/5 (0.0%) | 1/5 (20.0%) | 1/5 (20.0%) |
|  | Blood culture | 3/45 (6.7%) | -- | 0/45 (0.0%) | 1/45 (2.2%) | --- | --- | --- |
|  | Sputum | 9/61 (14.8%) | -- | 7/61 (11.5%) | 5/61 (8.2%) | 5/61 (8.2%) | --- | --- |
|  | BAL | 5/20 (25.0%) | -- | 5/20 (25.0%) | 2/20 (10.0%) | 2/20 (10.0%) | --- | --- |
|  | Bronchial secretion | 9/22 (40.9%) | -- | 5/22 (22.7%) | 2/22 (9.1%) | 2/22 (9.1%) | --- | --- |
|  | Liver biopsy | 1/1 (100.0%) | -- | 1/1 (100.0%) | 1/1 (100.0%) | 1/1 (100.0%) | 1/1 (100.0%) | 0/1 (0.0%) |
|  | GI biopsy | 1/10 (10.0%) | -- | 0/10 (0.0%) | 1/10 (10.0%) | 0/10 (0.0%) | 0/10 (0.0%) | 0/10 (0.0%) |
|  | Feces | 4/34 (11.8%) | -- | 4/34 (11.8%) | 0/34 (0.0%) | --- | --- | --- |
|  | Soft tissue | 4/10 (40.0%) | -- | 2/10 (20.0%) | 3/10 (30.0%) | 0/10 (0.0%) | 0/10 (0.0%) | 0/10 (0.0%) |
|  | Urine | 3/41 (7.3%) | -- | 3/41 (7.3%) | 0/41 (0.0%) | --- | --- | --- |
|  | CSF | 1/7 (14.3%) | -- | 1/7 (14.3%) | 0/7 (0.0%) | 0/7 (0.0%) | 0/7 (0.0%) | --- |
|  | swab | 7/15 (46.7%) | -- | 5/15 (33.3%) | 2/15 (13.3%) | --- | --- | --- |
|  | Lung biopsy | 1/1 (100.0%) | -- | 0/1 (0.0%) | 1/1 (100.0%) | 0/1 (0.0%) | 0/1 (0.0%) | 0/1 (0.0%) |
|  | Pleura biopsy | 5/9 (55.6%) | -- | 2/9 (22.2%) | 2/9 (22.2%) | 0/9 (0.0%) | 0/9 (0.0%) | 1/9 (11.1%) |
|  | Ascites | 1/3 (33.3%) | -- | 1/3 (33.3%) | 1/3 (33.3%) | 1/3 (33.3%) | 0/3 (0.0%) | --- |
|  | Others | 7/12 (58.3%) | -- | 3/12 (25.0%) | 4/12 (33.3%) | 1/12 (8.3%) | 2/12 (16.7%) | 3/12 (25.0%) |
|  | *GeneXpert/nested NTM-PCR | | | | | | | |

Table S2: Detected species by conventional methods in patients with diagnosed clinically relevant mycobacterial disease.

|  |  | **n/N (%)** |  |  |
| --- | --- | --- | --- | --- |
|  |  |  |  |  |
| **Diagnosed with TB** | | 59/74 (79.7%) |  |  |
|  | *M. tuberculosis* | 41/59 (69.5%) |  |  |
|  | *M. africanum* | 1/59 (1.7%) |  |  |
|  | *M. bovis* | 1/59 (1.7%) |  |  |
|  | *M. tuberculosis / M. xenopi* | 1/59 (1.7%) |  |  |
|  | No conventional identification | 19/59 (32.2%) |  |  |
|  | |  |  |  |
| **Diagnosed with NTM** | | 15/74 (20.3%) |  |  |
|  | MAC | 3/15 (20.0%) |  |  |
|  | *M. avium* | 3/15 (20.0%) |  |  |
|  | *M. intracellulare* | 1/15 (6.7%) |  |  |
|  | *M. genavense* | 2/15 (13.3%) |  |  |
|  | *M. chelonae / M. immonogenicum* | 1/15 (6.7%) |  |  |
|  | *M. genavense / M. avium* | 1/15 (6.7%) |  |  |

**Supplementary figures:**


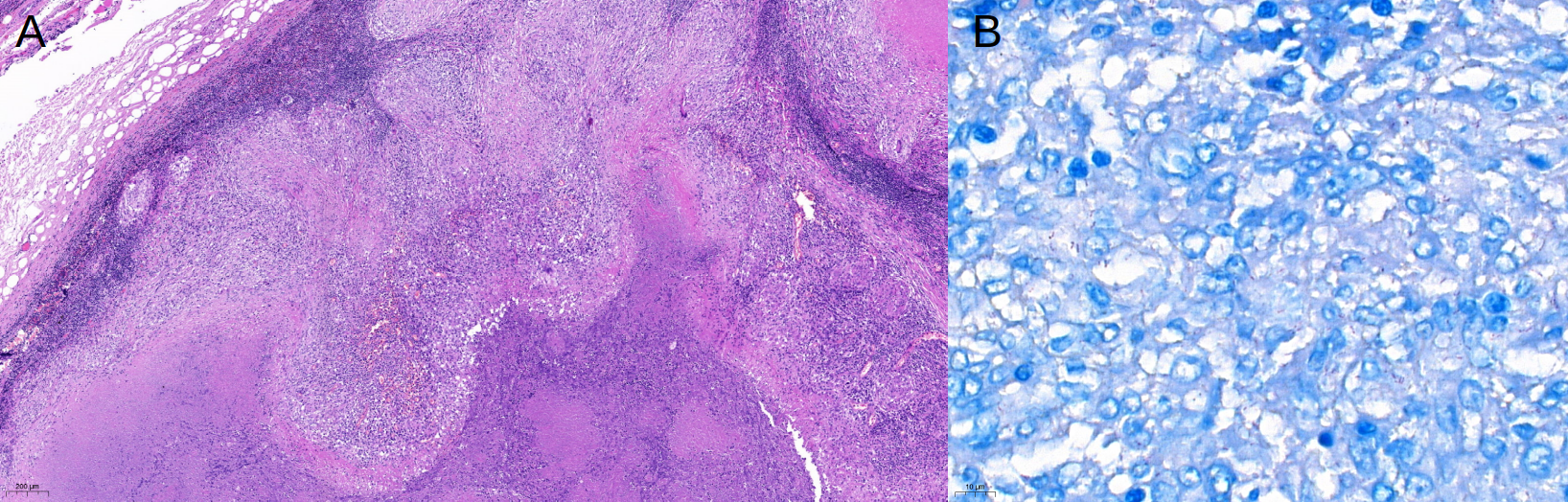
Figure S1: HE stained lymph node with caseating granulomas with central necrosis, epithelioid cell reaction and giant cells (A), Ziehl-Neelsen staining with mycobacterial infiltration in a lymph node (B).


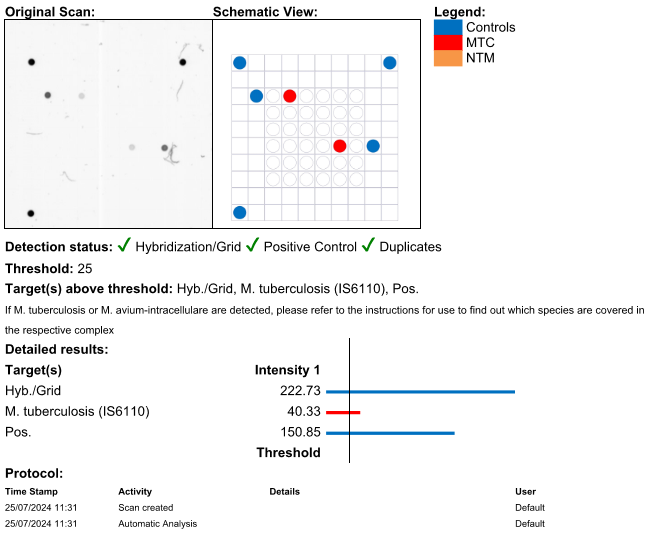


Figure S2: Example of a report with positive pathogen detection of *Mycobacterium tuberculosis*. MTC – *Mycobacterium tuberculosis* complex, NTM – non-tuberculous mycobacteria.


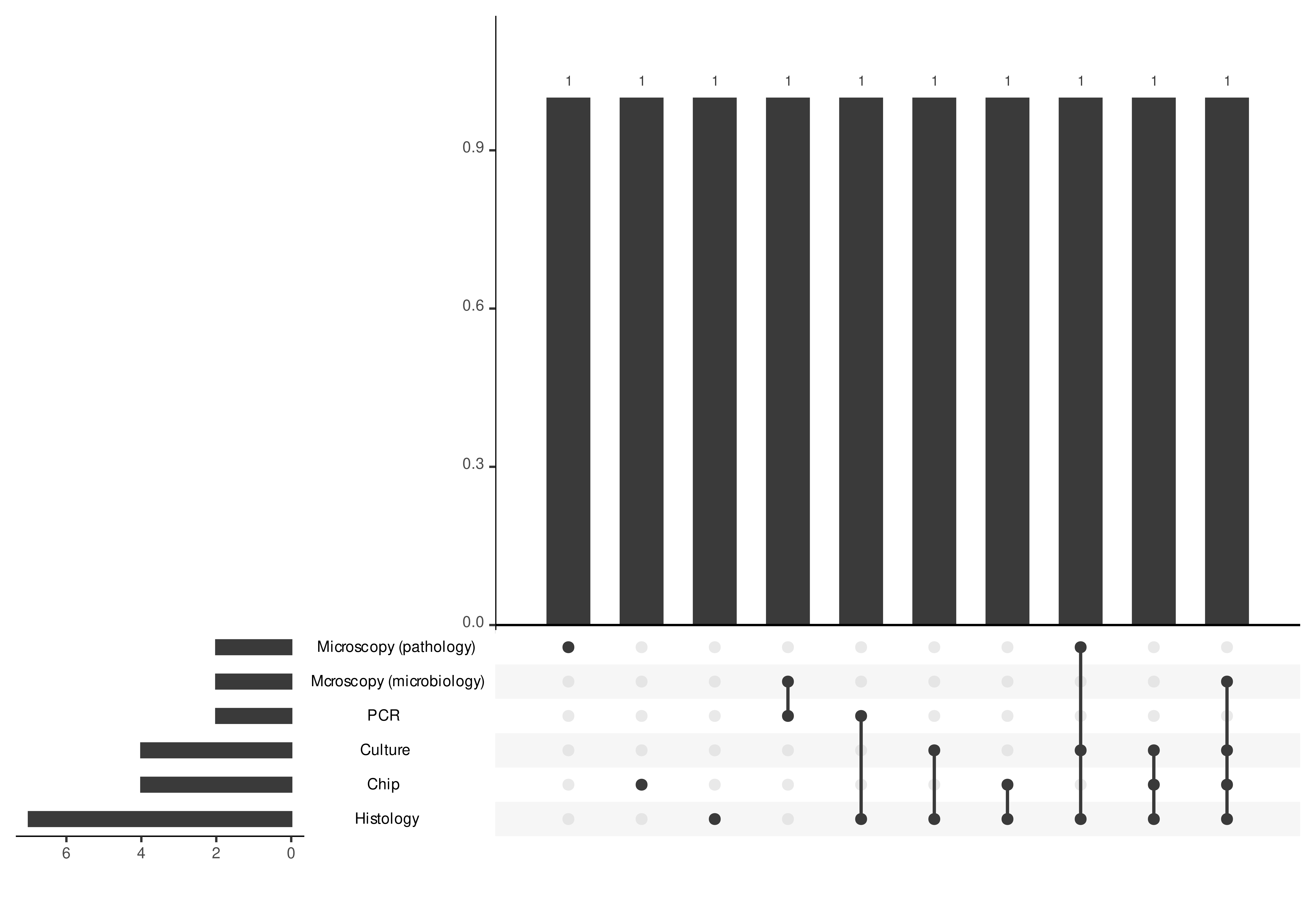
Figure S3: Upset plot of positivity of different diagnostic methods from the same material for patients with diagnosed NTM disease. Left panel shows overall number of samples that are positive by a diagnostic modality. Right upper panel shows the number of samples that are positive in a given combination of diagnostic modalities (right lower panel).
